# Supplementary material for: 3 versus 7 Tesla magnetic resonance imaging for parcellations of subcortical brain structures in clinical settings
Source: PLoS One. 2020 Nov 24;15(11):e0236208. doi: 10.1371/journal.pone.0236208 (PMC7685480; doi:10.1371/journal.pone.0236208)
Supplement: S1 File — (PDF) [file pone.0236208.s001.pdf]

3 versus 7 Tesla Magnetic Resonance Imaging for parcellations of subcortical brain structures

Bethany R Isaacs[1,2], Martijn J Mulder[1,3], Josephine M Groot[1], Nikita van Berendonk[1], Nicky Lute[1,4], Pierre-Louis Bazin[1,5], Birte U Forstmann[1]*, Anneke Alkemade[1]*

[1]. University of Amsterdam, Integrative Model-based Cognitive Neuroscience Research Unit, Amsterdam, The Netherlands

[2]. Maastricht University Medical Centre, Department of Experimental Neurosurgery, Maastricht, The Netherlands

[3]. University of Utrecht, Psychology and Social Sciences, Utrecht, The Netherlands

[4]. Vrije University, Clinical Neuropsychology, Amsterdam, The Netherlands

[5]. Max Planck Institute for Human, Cognitive and Brain Sciences, Leipzig, Germany

* Contributed equally

Corresponding author:

Anneke Alkemade, PhD
Integrative Model-based Cognitive Neuroscience
(IMCN) research unit
University of Amsterdam
Nieuwe Achtergracht 129B | Room G3.08
Postbus 15926 | 1001 NK Amsterdam
jmalkemade@gmail.com

Supporting Information 1: Anatomical descriptions

We have summarized the delineation procedures for the five tested structures:

1) The STN was parcellated on QSM images creating separate masks for each hemisphere in either fslview, or fsleyes (fsl.fmrib.ox.ac.uk). The STN was identified as a hyperintense structure anterior to the red nucleus superior to the dorsolateral extent of the SN. Raters first identified the ellipsoid shape of the STN by scrolling through the coronal slices. Parcellations were started at the central level of the STN, where it had its largest diameter in the coronal plane, and where the border with the SN was clearly discernable. The location of the STN/SN border was confirmed by visual inspection of the sagittal plane. Parcellations were performed in the coronal plane, and results were continuously checked in the sagittal and axial view for consistency. First the STN parcellations were continued in a caudal direction. After the disappearance of the STN, raters returned to their starting point, continuing parcellations in the rostral direction. Again, the size of the STN decreased, and after parcellation of the rostral extent of the STN the shape of the STN masks was checked in all three planes to confirm consistency. A final

check of the mask was performed using the 3D viewer tool. Finally all individual masks were stored, and used for further analyses.

2) The SN was parcellated on QSM images. The SN was identified in the coronal view, and also appeared as a hyperintense structure, using the globus pallidus and the mamillary bodies for anatomical orientation. Parcellations were started at the level where the STN had its maximum diameter and the border between the STN and SN was clearly discernable. First the border with the cerebral peduncle was delineated, and then the border with the STN. If the border with the STN was not clearly visible in a single slice due to partial volume effects with the SN, the slices anterior and posterior, as well as the sagittal and axial view were used to identify the likely border. The SN was first delineated in a caudal direction. The caudal extent of the SN in a subset of participants showed less hyperintense voxels in the SN, which we attributed to the nigrosomes. As the RN disappeared from the slices, the SN decreased in size. The caudal end of the SN was usually observed approximately 3 slices after the disappearance of the RN. Parcellations were continued in the anterior direction of the starting point. The rostral part of the SN bordered with the hyperintense GPi. At these levels the sagittal and axial view were checked to confirm the position of the border between these structures. After parcellation of the SN, the masks were checked in all three planes, and a final check was performed using the 3D viewer tool.

3) The RN is another iron-rich structure, and delineated on QSM contrasts. The RN was identified in the coronal plane. The parcellation was started at the level of the largest diameter in the coronal plane. The RN is a well-defined rounded structure which allowed a relatively easy delineation, which was performed first in a caudal and then in a rostral direction. In a subset of scans the fasciculus retroflexus (FR) was visible crossing through the red nucleus. In these cases the FR was included in the parcellations of the RN. After checking of all the mask in all three planes of view, the 3D viewer was used for a final check.

4) GPe delineation was started at the level where the caudate nucleus appeared as a separate structure from the putamen in the coronal view. The lateral border of the GPe was parcellated using the QSM contrast on which the GPe was hyperintense following the structure in a caudal direction. The GPe border to the striatum was difficult to discern at some levels on the QSM images, therefore the T1-maps were used to confirm the location of this border, and to adjust the mask if required. The same procedure was followed parcellating the border in the anterior direction. Borders that were difficult to discern in the coronal view were left unmasked, and subsequently drawn in the axial plane on the QSM contrast, and confirmed using the T1-maps. As a next step, The GPe was separated from the GPi by the medial medullary lamina (mml), which appeared as a hypotintense border in the QSM contrast. The mml was visualized using an overlay of the T1-map (50% transparency) over the QSM contrast, and the GPe border with the GPi was parcellated in the axial view. The rostral extent of the GPe was again parcellated in the coronal view. The dorsal border of the GPe was formed by the internal capsule, which appeared as a hypointense structure on the T1 map. The anterior commissure what used to define the

ventral border of the GPe. After checking of all the mask in all three planes of view, the 3D viewer was used to confirm the expected 3D shape of the GPe.

5) In the axial plane, the GPi was identified medial to the GPe on the QSM contrast. The GPi is delineated by starting at its most dorsal extent, and moving in a ventral direction. When the GPi started to become more difficult to delineate at a more ventral level, parcellation was continued in the coronal view. The incomplete medullary lamina was included as part of the GPi, and was visible in a subset of the participants.
