# Supplementary material for: 3 versus 7 Tesla magnetic resonance imaging for parcellations of subcortical brain structures in clinical settings
Source: PLoS One. 2020 Nov 24;15(11):e0236208. doi: 10.1371/journal.pone.0236208 (PMC7685480; doi:10.1371/journal.pone.0236208)
Supplement: Attachment — Submitted filename: rebuttal.docx [file pone.0236208.s002.docx]

**Comments to the Author**

**Reviewers' comments:**

Reviewer #1: Isaacs et al. compared the 3 and 7 Tesla magnetic resonance imaging (MRI) in visualizing the small brain structures, with the goal of utilizing the additional imaging information afforded by the 7T MRI to eventually replace the need of behavioral testing in awake patient during deep brain stimulation (DBS) surgery. The manuscript was clearly written, and the data presentation was well-organized.
My major concern is the lack of clarity in the authors’ conclusion. They set out to ask the question whether the 7T MRI represented a better option for imaging anatomical structures such as globus pallidus (GP) and subthalamic nucleus (STN). The data seemed to suggest that the answer was “it depends.” For imaging some larger structures, the 7T did outperform, while for some other structures, it did not. However, the conclusion reached by the authors was mainly about the importance of isotropic voxels. I would recommend the authors to stay focused on the main goal of this study when it comes to the conclusions, and clearly write about whether the 7T offers additional imaging information that could potentially improve surgical procedures and patient care, and if so, to what extent.

**Authors’ response:**

We would like to thank the reviewer for this excellent suggestion.

We have now rephrased the abstract focusing on our main conclusion (page 2, line 41-44):

*These results showed a higher consistency for structure volumes for 7T compared to optimized 3T which illustrates the importance of the use of isotropic voxels for 3D visualization of the surgical target area. Together these results indicate that 7T outperforms c3T as well as o3T.*

We have also rephrased the conclusion section (page 23, line 614-623):

*We set out to test whether 7T outperformed 3T MRI in the context of target visualization for DBS surgery. We now conclude that 7T outperforms 3T protocols. c3T protocols do not allow the rendering of biologically plausible 3D representation of small deep brain structures, they therefore cannot provide an accurate 3D account of the surgical area. o3T protocols using isotropic voxels strongly improved the imaging of the surgical area, although it was still outperformed by 7T imaging.*

**Reviewers' comments:**
Minor comments:
1. In the Results section of main text, under “Anatomical Distance” subtitle, when the authors reported the Bayes Factors (BFs) for GPi and STN, the interpretations seemed inconsistent with what the numbers indicated.

**Authors’ response:**

Thank you for pointing this out. We have now corrected these interpretations (page 15, line 373-376).

*When assessing for differences in distances across field strengths per structure, we found consistent evidence for no differences for the GPe (BF_01_ = 0.86, anecdotal), GPi (BF_01_ = 0.89, anecdotal), RN (BF_01_ = 0.32, substantial), SN (BF_01_ = 0.31, substantial), and STN (BF_01_ = 0.34, anecdotal).*

**Reviewers' comments:**

2. For Figure 2, please specify that the notations to the panels’ right are the voxel sizes.

**Authors’ response:**
We have now indicated in the legends that these are the voxel sizes (page 14, line 337):

*Voxel sizes are indicated on the right side of the figure.*

**Reviewers' comments:**

Reviewer #2: Summary and overall impressions

This manuscript describes the utility of higher resolution and ultra-high field MRI for anatomical segmentations of subcortical structures with clinical applications. In particular, the manuscript compares current clinical approaches using standard 3T MRI with optimized 3T MRI and with 7T MRI and how the acquisitions affect manual and semi-automatic basal ganglia segmentations. Overall, the manuscript provides solid evidence that 7T MRI enables improvements in segmentation of subcortical brain structures, and that isotropic voxel sizes are critical for accurate subcortical segmentation. On the whole, these results are not particularly surprising, but they are important for improving clinical imaging and establishing the clinical utility of ultra-high field anatomical MRI. Certain individual statistical results also suggest weaknesses in current MRI acquisition and semi-automated processing approaches, which are important to keep in mind, particularly for clinical investigations.

The data are appropriate for this investigation and are well-described. The results are similarly interpreted appropriately. While some additional control analyses would bolster the major claims of the manuscript, and I have a few minor concerns regarding the reporting of methods and results, I can recommend this manuscript for publication if they are addressed.

Larger concerns

There are a number of uncontrolled variables that could be influencing segmentation differences between acquisitions and parcellation schemes. Two major ones—resolution and acquisition sequence—are mentioned presently, although their effects are not fully addressed. Regarding resolution, which differs between the 7T and 3T acquisitions: a major result is the lack of CNR differences between 3T and 7T with semi-automated parcellation methods, as well as reduced segmentation consistency for smaller structures at 7T. To determine whether segmentation improvements are due to increased spatial resolution or signal contrast, it would be helpful to downsample the 7T data to the 3T resolution and compare it with both the native 7T resolution segmentations and the 3T segmentations. This downsampling would clarify the relative contributions of resolution and signal contrast.

**Authors’ response:**

We would like to thank the reviewer for his/her positive evaluation of our efforts.

Unfortunately, down sampling of the 7T data will affect not only the spatial resolution but also influence signal to noise ratios. Such control experiments will therefore not allow us to tease apart contributions of resolution and signal contrast. We have previously addressed the effect of spatial resolution by modeling of voxel size (Mulder et al., 2019). This modeling study provided information on the effect of increased spatial resolution, independent of potential contributions of signal contrast. We now refer to this studyin the discussion: (page 21, line 532-539).

*We have previously demonstrated the substantial impact of voxel geometry on parcellation accuracy* *[57]. It is therefore possible that our results using MIST may have underestimated the effects resulting from the higher spatial resolution that can be achieved using 7T. The largely absent CNR differences indicate that differences in spatial resolution can, at least in part, explain the differences observed using MIST. Down sampling of the 7T data may have led to an underestimation of the effect of the higher spatial resolution that was obtained from 7T scanning.*

**Reviewers' comments:**
Additionally, the manual and semi-automatic segmentations were performed on slightly different data: 3T manual segmentations were in native space, while 3T semi-automatic segmentations were on data warped to a common space. For the 7T data, manual segmentations were performed at native resolution, while semi-automatic segmentations were performed on (slightly) down sampled data. This last difference is particularly odd, given that at least one result in this manuscript is assigned to differences in resolution (3T vs. 7T small structure segmentation; Line 543) and needs controlling or further explanation.

**Authors’ response:**

This is an important issue, and we would like to thank the reviewer for pointing this out. MIST parcellations were attempted in native space, which resulted in anatomically implausible and small representation of anatomical structures. One possible explanation is that the MNI-prior did not provide enough information for smaller voxels. This problem was resolved by down sampling to 0.8 mm isotropic voxel size resolution.

We have now added this information to the Materials and Methods section. It now reads (page 7, line 211-213):

*MIST was unable to perform parcellations in 0.7 mm isotropic voxels, which we attributed to the limited information provided by the prior derived from MNI-space for these small voxels. Images were therefore resampled to 0.8 mm which resolved the problem.*

We hope that our additional explanation sufficiently clarifies why the data was warped and down sampled.

**Reviewers' comments:**
The manuscript should address what added value the quantitative maps provide over conventional weighted images. Ideally, this would include a comparison (at 3T and at 7T) between segmentations performed by T2-weighted images (from a single echo) and T2* maps. This would be useful data for deciding the utility of quantitative maps (over conventional weighted images) in clinical contexts.

**Authors’ response:**

We would like to thank the reviewer for pointing this out. The goal of our study was to investigate the added value of increased field strengths in clinical practice. To this end, we developed a scan protocol optimized for its potential use in a clinical setting. Our protocol allowed the calculation of quantitative contrasts. In view of their higher sensitivity to subtle global brain changes and their applicability within a clinical timeframe, we decided to use quantitative contrasts based on these theoretical benefits. We have now added a paragraph to the discussion (page 22, line 572-579):

*We developed a scan protocol optimized for its potential use in a clinical setting. Our protocol allowed the calculation of quantitative contrasts. In view of their higher sensitivity to subtle global brain changes, and their applicability within a clinical timeframe we decided to use quantitative contrasts. We did not compare quantitative MRI to conventional weighted MRI images, and we therefore cannot conclude that quantitative MRI positively influences parcellation results. However, the theoretical benefits of removing bias, and the potential application of quantitative MRI as a biomarker argues for the calculation of quantitative MRI contrasts.*

**Reviewers' comments:**

Despite a major take-home message being the importance of isotropic voxel sizes even at 3T, there is surprisingly no statistical comparison between the anisotropic (clinical) 3T scans and the isotropic (optimized) 3T scans. While not optimal, the anisotropic images do provide 3D data and could be segmented. There would likely be high variability between segmentations reflected in lower Dice scores than for the isotropic data, which would provide statistical evidence for the main conclusion of the manuscript.

**Authors’ response:**

We have considered creating parcellations of the c3T images and performing statistical comparisons. Since c3T parcellations did not yield any anatomically plausible shape of the structures of interest, we concluded that we could not provide any meaningful analyses based on such parcellations (see Figure). This is the reason we did not include them in our manuscript. To clarify further, it is conceivable that comparisons of Dice factors based on these parcellations could reveal a high level of interrater agreement. Performing statistical analyses on these data could then result in statistical differences. However, these differences would not reflect the visibility of the structure, since the parcellation itself does not have anatomical relevance, unlike those obtained by o3T and 7T parcellations.

Figure illustrating STN parcellation results in the clinical 3T images. On the left the parcellated images. On the right the resulting, anatomically implausible 3D rendering of the STN.

We have now clarified this in the manuscript (page 20, line 461-496):

*We considered delineating the individual brain structures on c3T images, and performing statistical comparisons. However, c3T parcellations did not yield anatomically plausible renderings of these structures. It is conceivable that these results would have revealed a high level of interrater agreement, as well as statistically significant differences. However, given the lack of anatomical relevance of these parcellations we deemed such analyses valueless.*

**Reviewers' comments:**

Minor concerns
Regarding the presentation of statistical results, there are many comparisons made in this manuscript, each of which is appropriate. However, the tables could be difficult to read with confidence. These issues likely reflect my relative unfamiliarity with Bayesian statistics, but other readers may have similar interpretation issues, so addressing these concerns is important.
For example, noting which comparisons use one-tailed or two-tailed tests in the tables would be very helpful, as it’s currently challenging to understand the direction/interpretation of effects at first glance. Perhaps separating or delineating the tables based on one-tailed vs. two-tailed tests would be easier to interpret.

**Authors’ response:**

We would like to thank the reviewer for this important and constructive comment. We have made the required adjustments to the tables. We have indicated in Table 2 that Dice scores and CNR were tested using one-tailed paired samples t-tests, and in the legend of Table 3 we have indicated that all comparisons were performed with two-tailed paired samples t-tests.

**Reviewers' comments:**

In general, the tables are conveying a lot of statistical information but can be difficult to parse. For instance, in Table 3b, it is unclear where “Anatomical Distance” measures end and “QSM CNRs” begin. Extending the vertical separators to the highest level would help.

**Authors’ response:**

We have extended the separators in all results tables according to the reviewer’s suggestion and increased the spacing, which has made the table layout easier to parse.

**Reviewers' comments:**

Further, despite relying on quantitative mapping paradigms, there is limited discussion of conventional “weighted” images vs. quantitative maps. The manuscript should discuss rationale for using maps and explain how they differ from conventional images. Incorporating a color bar in the figures (showing correspondence between image intensity and physical units) would help readers intuit the “quantitativeness” of T1 and T2* maps.

**Authors’ response:**

We agree with the reviewer that this topic is both important and interesting. However, our goal was not to demonstrate the added value of quantitative contrasts over conventional weighted images. We have clarified this above, and hope that the reviewer agrees that we have adequately discussed the importance of this topic in the Discussion section (page 22, line 572-579):

*We developed a scan protocol optimized for its potential use in a clinical setting. Our protocol allowed the calculation of quantitative contrasts. In view of their higher sensitivity to subtle global brain changes, and their applicability within a clinical timeframe we decided to use quantitative contrasts. We did not compare quantitative MRI to conventional weighted MRI images, and we therefore cannot conclude that quantitative MRI positively influences parcellation results. However, the theoretical benefits of removing bias, and the potential application of quantitative MRI as a biomarker argues for the calculation of quantitative MRI contrasts.*

**Reviewers' comments:**
Line-specific comments
Line 142: Inconsistent reporting of TA/total acquisition time across data acquisitions in the methods (here “ms”, usually “min”). As “TA (Acquisition Time)” is ambiguous and can refer to active volume collection within a TR in a sparse fMRI design, something like “scan duration/length” or “total acquisition time” would be clearer.

**Authors’ response:**

We corrected ms to min. Additionally, we have checked the use of TA for consistency. TA is now reported as the time needed to acquire the T1 and T2-weighted images, the total acquisition time refers to TA_T1_ + TA_T2_ for the 3T scans, and for the 7T TA is the same total acquisition time since images are obtained from a single scan acquisition.

**Reviewers' comments:**

Line 163: A bit more description of T1 map estimation would be helpful. In particular, T1 map estimation from the 3T data, as the o3T T1-weighted image only has one data point. Citations of previous uses of this mapping would also be beneficial. This paragraph should also be under a “Image processing” (or similarly named) section, not under “Data acquisition.”

**Authors’ response:**

We apologize for the confusing wording: map estimation for the o3T data was only for the T2* map, while the T1 contrast remains T1-weighted for both o3T and c3T. We have now created a heading “Image Calculations”, and clarified the computations (page 6, line 160-164).

*T2* maps for o3T and 7T MRI scans were created by least-squares fitting of the exponential signal decay over the multi-echo images of the second inversion. 7T T1-weighted images were generated by complex ratio of the product of first and second inversion over the sum of their square [37]. A quantitative T1 map was also reconstructed from this T1-weighted image via a look-up table procedure [37].*

**Reviewers' comments:**

Line 194: “QSM-maps and T1-images were used as input for MIST.” T1-weighted or T1 maps? It sounds like T1 maps but please clarify (but also see concern re: Line 163).

**Authors’ response:**
MIST was designed to use T1-weighted images and cannot perform parcellations using the T1-maps instead. We have now corrected T1-images to T1-weighted images.

**Reviewers' comments:**

Line 214: Why resample to 0.8 mm? If isotropic voxel size is required, why not 0.7 mm? Additionally, right now the manual segmentation used full resolution (0.64x0.64x0.7), but MIST used 0.8 mm. To directly compare segmentation methods, statistical comparisons should be conducted on the same input data.

**Authors’ response:**

Resampling was performed to accommodate limitations in the MIST parcellation approach. We have explained this on page 7, line 211-213, which now reads:

*MIST was unable to perform parcellations in 0.7 mm isotropic voxels, which we attributed to the limited information provided by the prior derived from MNI-space for these small voxels. Images were therefore resampled to 0.8 mm which resolved the problem.*

**Reviewers' comments:**

Line 232: Anatomical distance is computed as the center of mass between left and right hemisphere structures and is therefore sensitive to segmentation quality in each structure. Therefore, a center-of-mass difference in one hemisphere’s structure (when comparing two different scans/segmentation methods) could be masked by an equal but opposite center-of-mass difference in the other hemisphere’s structure. This is minor, and there aren’t indications that it dramatically affects the overall interpretation of results, but it should be addressed by using a control reference point (such as midline posterior commissure), or at least discussed.

**Authors’ response:**

We have now clarified that we expected that altered visibility of specific borders of a structure would be reflected in the anatomical distance. It is possible that an effect could be masked by an equal but opposite difference, however, determination of an independent reference point, such as the midline posterior commissure could potentially also vary between field strengths. We therefore decided not to include such a variable. We have added this information to the manuscript (page 20, line 509-513):

*If the changes in volume were the result of altered visibility of a specific anatomical border, this would result in a position shift of the center-of-mass. Since such a shift would be present in both hemispheres, we expected it to be reflected in a change in anatomical distance. It is important to note that it is possible that an equal but opposite effect in the other hemisphere could obscure shifts the center-of-mass.*

**Reviewers' comments:**

Line 276: “Preferred model” could use clarification. Is it just the model with greater evidence (logarithmic distance from 1)? Tables 3a and 3b are also ambiguous in denoting the preferred model.

**Authors’ response:**

We have now checked the tables and clarified (page 10, line 275-276) that the preferred model refers to the model for which highest evidence is present based on model comparisons:

*The preferred model, which was the model for which strongest evidence was present, was then reported along with the model comparisons.*

**Reviewers' comments:**

Line 280: For readers who are less familiar with Bayesian statistics, it would be helpful to describe differences in BF reporting between one-tailed and two-tailed tests: for one-tailed, BF > 1 suggests M1 (group 1 > group 2), while BF <1 suggests M2 (group 2 > group 1). Whereas for two-tailed, BF > 1 suggests a difference between groups without preferring one group over the other, while BF < 1 suggests no difference between groups.

**Authors’ response:**

We would like to thank the reviewer for this suggestion. We have made the required adjustments to the tables. We have indicated in Table 2 and 3 whether one-tailed or two-tailed tests were performed.

**Reviewers' comments:**

Line 302: At present, the data and processing scripts are hosted on OSF but are not available without requesting access. Please make the project data and scripts public for inspection during the review process.

**Authors’ response:**

We have now adjusted the settings so that the data on OSF are freely available without restrictions.

**Reviewers' comments:**

Table 2: QSM CNRs – GPe: o3T > 7T, but BF is described as “M_2 BF_10”. If model 2 is preferred, shouldn’t BF be reported as BF_01? (according to the caption) However, then the BF should be < 1.

**Authors’ response:**

We have corrected this mistake.

**Reviewers' comments:**

Line 370: GPi (BF = 0.89) and STN (BF = 0.34) should be “anecdotal”, not “substantial” (per Bayes Factor Interpretation cut-off values in Table 1).

**Authors’ response:**

We have now corrected this mistake and thank the reviewer for pointing it out.

**Reviewers' comments:**

Line 398: Similar to Line 280, more specific statistical language would be helpful (at least for the first description of two-tailed results). For instance, could say “For o3T we found significant evidence for a difference between manual and MIST segmentations of GPe/i and STN, with both being smaller with MIST than with manual parcellations.”

**Authors’ response:**

We have checked and rephrased the text (page 15, line 401-403):

*For o3T, resulting MIST parcellation volumes were smaller than those resulting from manual parcellations for GPe/i (BF_10_ = 456.18, decisive) and STN (BF_10_ = 11.96, strong).*

**Reviewers' comments:**

Line 424: STN should be reported as BF_10, not BF_01, as per Table 3b and the present interpretation.

**Authors’ response:**

We have corrected this mistake.

**Reviewers' comments:**

Table 3a: It’s ambiguous what the “preferred model” is, as the “**” annotation seems to be pointing to a statistical test, not to a specific model.

Table 3b: Same “**” preferred model issue – can’t tell which model is preferred.

**Authors’ response:**

We have changed the notation to numbers to remove the confusion and clarified in the notes which model is the preferred model.

**Reviewers' comments:**

Line 525: Re: segmentation method differences: address possible contributions of data resampling prior to MIST but not manual segmentation (resulting in different resolution, along with potential partial voluming effects and SNR changes).

Line 544: CNR vs. resolution could be assessed by downsampling 7T data to 3T resolution and comparing segmentations with high res 7T, o3T segmentations

**Authors’ response:**

We have added a paragraph to the discussion clarifying these points (page 21, line 532-539):

*We have previously demonstrated substantial impact of voxel geometry on parcellation accuracy* *[57]. It is therefore possible that our results using MIST may have underestimated the effects resulting from the higher spatial resolution that can be achieved using 7T. The largely absent CNR differences indicate that differences in spatial resolution can, at least in part, explain the differences observed using MIST. Down sampling of the 7T data may have led to an underestimation of the effect of the higher spatial resolution that was obtained from 7T scanning.*

We have made adjustments to the tables. We have indicated in Table 2 that Dice scores and CNR were tested using one-tailed paired samples t-tests, and in the legend of Table 3 we have indicated that all comparisons were performed with two-tailed paired samples t-tests.

**Reviewers' comments:**

Line 554: Lack of improvement at 7T could also be due to sequence differences – are there downsides to the combined T1w/T2w MP2RAGEME sequence? Does the lack of field inhomogeneity correction contribute?

Line 561: first mention of field inhomogeneity. Given that Caan et al. (2019) say that “Good B1+‐inhomogeneity correction of both MP2RAGE and MP2RAGEME data is essential for successful cortical segmentation using automated routines (Haast, Ivanov, & Uludağ, 2018)”, this manuscript should address its lack of field correction and the potential effects on subcortical segmentation.

**Authors’ response:**

The lack of improvement referred to relates to the surgical targeting, which is to a large extent dependent on decisions made by the neurosurgical team. We have now clarified this in the manuscript (page 22, line 567-569):

*We cannot conclude whether this lack of improvement can be attributed to the MRI imaging or is the result of other factors, including the variation between surgeons* *[66].*

And page 22, line 588-592:

*In the MP2RAGEME, B0 inhomogeneities are automatically cancelled through the use of a ratio image* *[70]. Additionally, the subcortical parcellations presented in the current studies are largely dependent on grey matter contrasts, and we optimized contrast using flip angles of 4/4 instead of 7/6 degrees.*

**Reviewers' comments:**

Line 567: If further analyses are not conducted, need to add other limitations (differences in voxel sizes, sequences, field inhomogeneities, etc.).

**Authors’ response:**

We have now clarified that our studies should not be considered to reflect the limits of the 3T and 7T systems in a research setting, but rather as their limits within a clinical framework (page 23, line 619-623).

*The constraints posed by the clinical applicability of the imaging protocol contributed to limitations including differences in voxel sizes, scan sequences, field homogeneity. The results presented in the current studies should therefore be interpreted within the clinical framework, as they are not an account of the limits of 3T and 7T imaging within* *a research setting.*

**Reviewers' comments:**

Figure 1: Left column (T1-related) images need specific subtitles. 7T “MP2RAGE” – is it T1-weighted (UNI) or T1 map? Optimized 3T: sounds like it’s a “T1w” image, but figure suggests it’s also MP2RAGE.

**Authors’ response:**

We have now clarified that these are all T1-weighted images, both in the caption and the figure annotations.

**Reviewers' comments:**

Figure 3: It would be helpful to see an unsegmented QSM image next to the segmented ones so that readers can visually assess segmentation performance. If space is an issue, perhaps one side half of the brain could be unsegmented and one half could be segmented.

**Authors’ response:**

We have now added an extra Figure (now Figure 3) illustrating the manual STN delineation, and illustrating the unlabeled QSM figure.

**Reviewers' comments:**

Reviewer #3: This manuscript has not been prepared carefully and many errors and inconsistencies in formatting need to be corrected (see below). The use of vendor-specific acronyms for pulse sequences and parameters in the Methods section is very difficult to follow without a vendor specific manual.

**Authors’ response:**

We apologize for the inconvenience and have carefully checked the manuscript for formatting errors and vendor specific parameters.

**Reviewers' comments:**

Therefore, a full evaluation of differences in the selection of methods and parameter is not possible by the reader but should have been supplied by the authors. The c3T, o3T and 7T methods differ is so many parameters that comparing the results may not address the stated goal of the study. In the Discussion, the following statement illustrates the fundamental weakness of this comparison, “It is important to note that technically it would be possible to match the o3T scan sequence more closely to the 7T sequence. However, the resulting protocol would not meet the criteria for implementation in clinical practice due to SAR and time constraints. It is thus important to note that the improved performance of the 7T protocol cannot be exclusively attributed to differences in field strengths, but also to the constraints imposed by clinical practice.”

Greater effort is necessary to adequately prepare comparable acquisition methods on the 3 T and 7 T systems to allow significant conclusion to be drawn. Also, Reference 31 demonstrates the advantage of isotropic resolution available using higher fields, like 7 T, which is a stated in the Conclusion section as a main result.

**Authors’ response:**

We fully agree with the reviewer that the study is limited to investigate whether 7T can outperform 3T within the constraints of clinical practice. To be fully transparent and to emphasize this point, we have now changed the title to:

*3 versus 7 Tesla Magnetic Resonance Imaging for parcellations of subcortical brain structures in clinical settings*

We also added this information to the abstract (page 2, line 41-44):

*Together these results indicate that 7T outperforms c3T as well as o3T given the constraints of a clinical setting.*

We would like to point out again that we did not set out to provide a direct comparison on the limits of performance of the 3T and 7T systems, but on their limits within a clinical setting. We now emphasize that within a clinically feasible timeframe, the 7T outperforms standard clinical 3T acquisition protocols. Another important finding is that within the clinical setting, using 3T MRI imaging can be improved further.

**Reviewers' comments:**

Keywords should include 7 Tesla

**Authors’ response:**

7 Tesla has been added to the keywords.

**Reviewers' comments:**

The chosen referencing system (names versus numbers) is not applied consistently.
Error is punctuation, e.g. missing period at the end of the sentence on line 72.
Units should be separated from numerical values (i.e. 3T, 8.2ms), but this format is not followed consistently.
On line 142, the TA units are not correct.

**Authors’ response:**

We have corrected these points.
